# Restructuring Skin Cancer Care in Ontario: A Provincial Plan

**DOI:** 10.3390/curroncol28020114

**Published:** 2021-03-12

**Authors:** John Milkovich, Tim Hanna, Carolyn Nessim, Teresa M. Petrella, Louis Weatherhead, An-Wen Chan, Jonathan C. Irish, Christian Murray, Grace Bannerman, Claire Holloway, Katharina Forster, Laura Pazzano, Frances C. Wright, the Ontario Skin Cancer Advisory Committee

**Affiliations:** 1 Surgical Oncology Program, Ontario Health-Cancer Care Ontario, Toronto, ON M5G 2L7, Canada; milkovii@mcmaster.ca (J.M.); jonathan.irish@uhn.on.ca (J.C.I.); 2 Division of Cancer Care and Epidemiology, Cancer Research Institute at Queen’s University, Kingston, ON K7L 3N6, Canada; Tim.Hanna@kingstonhsc.ca; 3 Department of Oncology, Queen’s University, Kingston, ON K7L 5P9, Canada; 4 Division of Dermatology and Medical Oncology, The University of Ottawa, Ottawa, ON K1H 8L6, Canada; cnessim@toh.on.ca (C.N.); abedweatherhead@yahoo.ca (L.W.); 5 Sunnybrook Health Sciences Centre, Department of Medical Oncology, Toronto, ON M4N 3M5, Canada; teresa.petrella@sunnybrook.ca; 6 Department of Medicine, Women’s College Hospital, Toronto, ON M5S 1B2, Canada; An-Wen.Chan@wchospital.ca; 7 Department of Medicine, University of Toronto, Toronto, ON M5S 1B2, Canada; Christian.Murray@wchospital.ca; 8 Clinical Institutes and Quality Programs, Ontario Health, Toronto, ON M5G 2L7, Canada; grace.bannerman@ontariohealth.ca (G.B.); claire.holloway@ontariohealth.ca (C.H.); katharina.forster@ontariohealth.ca (K.F.); laura.pazzano@ontariohealth.ca (L.P.)

**Keywords:** skin cancer, pathway map, Merkel cell carcinoma, melanoma, basal cell carcinoma, squamous cell carcinoma, keratinocyte carcinoma, organization of care, management plan

## Abstract

There is a global rise in skin cancer incidence, resulting in an increase in patient care needs and healthcare costs. To optimize health care planning, costs, and patient care, Ontario Health developed a provincial skin cancer plan to streamline the quality of care. We conducted a systematic review and a grey literature search to evaluate the definitions and management of skin cancer within other jurisdictions, as well as a provincial survey of skin cancer care practices, to identify care gaps. The systematic review did not identify any published comprehensive skin cancer management plans. The grey literature search revealed skin cancer plans in isolated regions of the United Kingdom (U.K.), National Institute for Health and Care Excellence (NICE) guidelines for skin cancer quality indicators and regional skin cancer biopsy clinics, and wait time guidelines in Australia and the U.K. With the input of the Ontario Cancer Advisory Committee (CAC), unique definitions for complex and non-complex skin cancers and the appropriate cancer services were created. A provincial survey of skin cancer care yielded 44 responses and demonstrated gaps in biopsy access. A skin cancer pathway map was created and a recommendation was made for regional skin cancer biopsy clinics. We have created unique definitions for complex and non-complex skin cancer and a skin cancer pathways map, which will allow for the implementation of both process and performance metrics to address identified gaps in care.

## 1. Introduction

Skin cancer is the most common type of cancer in Canada. In 2019, 7300 new cases of melanoma were reported; however, there is a paucity of reported data for epidermal-derived skin cancer (keratinocyte carcinoma (KC)), where cutaneous squamous cell carcinoma (SCC) and basal cell carcinoma (BCC) are the predominant types [1]. In 2014, the Canadian Cancer Statistics focused on skin cancers and reported 76,100 new cases of KC [2]. Importantly, data from Canada and other countries suggest that skin cancer incidence is increasing [2,3]. As a result of its ubiquity, the healthcare costs for skin cancer care are significant and important to quantify for health care planning and optimal patient care. Recent estimates show that the annual costs associated with skin cancer in Canada amount to $532 million, and by 2031, it is expected that the annual cost will reach $922 million [4].

With its rising incidence and associated healthcare costs, it is important to implement efficient patient-centered care processes for skin cancer patients. Recent studies have identified that better organization of skin cancer clinical care could benefit Ontario residents. In a paper written by Look Hong et al., it was demonstrated that patients diagnosed with melanoma who only saw a family physician were more likely to receive an inadequate wide local excision of their primary lesion [5]. In another study, Ontario residents diagnosed with melanoma and who lived in rural communities or long distances from a cancer center were less likely to be seen by a dermatologist within a year before their diagnosis. This was related to a significantly higher risk of presenting with stage III or IV melanomas [6].

Ontario Health is the provincial agency that is responsible for overseeing health care delivery, improving clinical guidance, and supporting providers to ensure quality care for the 14.7 million people in the province of Ontario, Canada [7]. At the time of this work, Ontario was subdivided into 14 Local Health Integration Networks (LHINs), each with a Regional Cancer Program (RCP) led by a Regional Vice President (RVP). The RVP is responsible for organizing local cancer care to meet the standards and requirements set by Ontario Health (Cancer Care Ontario) (OH (CCO)). To identify specific disease site issues provincially, OH (CCO) has also implemented a disease site approach supported by the Disease Pathway Management (DPM) program. There are Ontario Cancer Leads (OCLs) and Cancer Advisory Committees (CACs) for each disease site with a mandate to identify disease-site-specific issues and improve quality of care [8]. The OCLs and CACs develop improvement plans with OH (CCO)’s Provincial Program Heads and Clinical Leads (i.e., Surgical Oncology, Systemic Treatment, Diagnostic Imaging), who are accountable for leading quality and performance management activities with the RCPs. For skin cancer, the OCL (F.C.W) and the Skin CAC identified the need for a centrally organized skin cancer care plan. In this paper, we describe the current state of skin cancer management in Ontario and the process of creating high-level planning tools to address the identified gaps in care. 

## 2. Materials and Methods

The process included a systematic and grey literature review, a provincial survey of skin cancer care practices, the creation of complex and non-complex skin cancer definitions, a description of appropriate clinical services, the formation of a skin cancer pathways map, and a recommendation for regional skin cancer biopsy clinics.

### 2.1. Literature Review

A systematic literature search was conducted by a librarian for primary evidence (original research, clinical case studies, and clinical trials) regarding the standards of care and treatment approaches for skin cancer that was published between January 2015 and November 2018. Using a comprehensive two-step search strategy, the search was executed in Ovid MEDLINE on November 9, 2018. The first set of keywords and Medical Subject Heading (MeSH) terms of searches focused on the clinical characteristics of complex and non-complex skin cancer, while the second set focused on the standard of care and treatment, with both sets using relevant search terms (see Appendix A for the full search strategy). Non-English sources were excluded from the literature search. A subsequent grey literature search was completed to identify skin cancer treatment organization that was not published in the medical literature.

### 2.2. OH (CCO)’s Skin Cancers Advisory Committee Discussions 

In September of 2018, the Skin CAC, comprised of skin cancer experts (dermatology, surgeons, medical and radiation oncology, primary care, and pathology) from across the province discussed the Ontario skin cancer epidemiologic data and the results of the literature search on the organization of skin cancer services in other jurisdictions. It was determined that the planning and organization for skin cancer services in Ontario could be facilitated by categorizing skin cancers as complex or non-complex since the two patient populations are distinct and require different clinical services and different levels of multidisciplinary care. Several subsequent meetings were held to collate further expert feedback on the definitions and clinical services required.

Additionally, the CAC recommended developing a skin cancer pathways map to foster a provincially standardized and evidence-based approach to diagnosing and treating complex and non-complex skin cancers. Initially, the pathways map’s development began in a small working group from the Skin CAC, followed by a subsequent review with the entire Skin CAC. Next, an internal review by provincial clinical heads in Surgical, Medical and Radiation Oncology, and Patient Support and an external review (circulated via RVPs to a broader group of stakeholders involved in skin cancer care within each region) were completed. Feedback was incorporated and the final version of the pathway map was reviewed and formally endorsed by the Skin CAC and OH (CCO) provincial clinical heads. The pathways map was designed to apply to 80% of skin cancer clinical scenarios.

### 2.3. Provincial Assessment of Access to Skin Cancer Care

To engage and receive feedback from regional partners, the Provincial Leadership Council (RVPs from a Regional Cancer Centre (RCC) in each LHIN) met to discuss skin cancer epidemiology in Ontario, as well as initial definitions of complex and non-complex skin cancers and their associated service requirements. Support was garnered for a provincial survey addressing the access to skin cancer services, which was developed by the OCLs with input from the Skin CAC, piloted with two RVPs, and then adjusted accordingly. In April 2019, the survey was delivered electronically to the RVPs, who distributed it throughout their LHIN. Two reminders were sent electronically to complete the survey before its closure in June 2019. The responses were summarized and re-presented at the Provincial Leadership Council in October 2019.

## 3. Results

### 3.1. Literature Review for Skin Cancer Organization of Care 

The systematic literature review identified nine papers, none of which described a regional organization of skin cancer services. A subsequent grey literature search identified stand-alone skin cancer clinics and guidelines for the clinical care of patients with melanoma and KC in Australia, organizational work in one region of the National Health Service (NHS) defining KCs as low and high risk, and seven quality metrics for skin cancer care in the U.K. [9,10,11,12,13]. A key finding from the NHS and Australia was the standardization of wait times. The NHS recommends that a patient with urgent skin cancer be seen by a specialist within 14 days of receiving a referral. From the date of diagnosis, a maximum 31-day wait time for definitive treatment is mandated [13]. Similarly, Australia’s National Cancer Expert Reference Group (NCERG) recommends a 2-week timeline for a skin biopsy performed by a primary care provider and for a specialist appointment as part of an optimal care plan [9].

### 3.2. Definitions of Complex and Non-Complex Skin Cancers and Clinical Services

Using the literature review and the Skin CAC’s expert opinion, it was decided to define and categorize skin cancers as complex and non-complex to facilitate the provincial organization of skin cancer care (Table 1). Patients were considered to have complex skin cancers based on patient factors (inoperable or skin cancer associated with a genetic syndrome), tumor factors (node-positive, locally advanced, metastatic, mucosal/ocular subtypes, in-transit/satellite lesions or recurrences), and treatment factors (patient requires a lymph node dissection or resection of metastatic disease, a medical or radiation oncology opinion, multidisciplinary care, access to clinical trials or Mohs micrographic surgery (MMS)). Factors particular to each histologic type of skin cancer (melanoma, Merkel cell carcinoma, BCC, or SCC) were also included. The appropriate services were then enumerated (Table 2). In general, complex skin cancer diagnoses are to be referred to an RCC or an MMS center, whereas non-complex skin cancer diagnoses can be treated in the community outside of an RCC [14]. 

### 3.3. Provincial Survey Regarding Access to Skin Cancer Care

At least one complete survey was received from each LHIN, with a total of 44 responses that illustrate the access to skin cancer care services (Appendix B). Notably, the qualitative data showed variations in the timely access to dermatology and surgery for diagnosis; a lack of centralized or coordinated access to diagnostic biopsy and treatment; varying degrees of access to dermatology, diagnostic biopsy, operating room resources, PET scans, and MMS. As a result of these findings, the Skin CAC recommended that regions adopt a similar structure to Australia’s skin cancer clinics by offering skin biopsy training to primary care providers and/or by developing skin cancer biopsy clinics. One LHIN has piloted a skin biopsy clinic, where this model is now available to the other LHINs. 

### 3.4. Development of a Skin Cancer Pathways Map

The Skin Cancer Pathways Map provides an overview of the skin cancer care continuum in the form of a flowchart, starting from the initial presentation and diagnosis and branching into five unique treatment plans that are specifically tailored to the histologic type of skin cancer and whether it is considered complex or non-complex (benign, Merkel cell carcinoma, melanoma, BCC, SCC). A key feature of the Skin Cancer Pathways Map includes distinct targeted wait times for skin biopsies for pigmented and non-pigmented skin lesions (2 weeks wait time and 4 weeks wait time, respectively). Although preferred, if the primary care provider is unable to provide a timely biopsy or if a biopsy requires a specialist (e.g., a subungual or periorbital lesion), it is recommended that a surgeon, plastic surgeon, dermatologist, otolaryngology/head and neck surgeon, or ophthalmic plastic surgeon perform the skin lesion biopsy https://www.cancercareontario.ca/en/pathway-maps/skin-cancer accessed on 8 March 2021 [15]. In addition to highlighting the best practices across the care continuum, the pathway development process helped to identify gaps in the current guidelines and evidence, particularly for Merkel cell carcinoma and KCs.

## 4. Discussion

We have described the development of a comprehensive health system plan for skin cancer care in Ontario. First, we defined complex and non-complex skin cancer and determined the required clinical services to provide optimal care. We then developed the Skin Cancer Pathways Map, which outlines the best practices for diagnosis and treatment. This work is unique as it defines skin cancers by the complexity of the clinical services required for appropriate treatment and encompasses a pathway for all aspects of skin cancer care. A key finding from our Skin CAC and the provincial survey was the lack of organization and, to a lesser extent, capacity (personnel to organize the clinics) around the diagnosis and treatment of skin cancer. Since then, we have initiated the implementation of a provincial plan for regional skin cancer biopsy clinics. 

In Australia, skin cancer clinics dispersed throughout the country provide patients with greater access to skin checks, mole removal, skin biopsies, skin cancer surgery, cryotherapy, and non-surgical treatments in a primary care setting and do not require a referral from a GP [16,17]. Regarding the relative diagnostic accuracies of excised or biopsy skin lesions by regular GPs versus skin cancer doctors in Queensland, Australia, both demonstrated high levels of diagnostic sensitivity for any skin cancer, including SCC, BCC, and melanoma [18]. It should be noted that not all primary care physicians are comfortable performing skin biopsies. To address this potential issue, the Skin CAC recommends that each LHIN develops a skin cancer diagnosis process by offering primary care providers skin biopsy training and/or by developing centralized skin cancer biopsy clinics. One LHIN (North Simcoe Muskoka, Barrie, Ontario, Canada) has already developed such a clinic, offering patients ready access to a dermatologic assessment of skin lesions for suspected malignancy and timely and appropriate referrals to a multidisciplinary network of specialists at the RCC. In this clinic, family medicine residents work with the dermatologist to learn how to assess skin lesions and perform skin biopsies.

Clinical pathways delineate evidence-based, locally feasible, stepwise plans that outline multidisciplinary care for distinct populations of patients [19]. Internationally, streamlined cancer pathways have been used to close gaps in the delivery of care, reduce healthcare costs and wait times, and improve patient outcomes [20,21,22,23,24,25]. The primary functions of the DPM’s cancer pathway maps are to promote evidence-based best practice and to provide Ontario’s health care providers with a high-level overview of the treatment plan [26]. Notably, the establishment of a specified time to biopsy (2 weeks for pigmented or rapidly growing lesions and 4 weeks for non-pigmented lesions) and the recommendations for preferred providers to make the diagnosis (primary care provider) are distinct features of the Skin Cancer Pathways Map that promote timely care. These align with the third statement of the National Institute for Health and Care Excellence (NICE)’s seven quality standards regarding skin cancer care, stating that suspected melanoma cases should be referred to a specialist for diagnosis using a suspected cancer pathway for an appointment within 2 weeks [12]. Importantly, the NHS reported that 92.6% of 469,575 patients with suspected skin cancer were seen by a specialist within the 2-week wait time standard [27]. From referral to definitive treatment, in the U.K., it is mandated that a patient with a malignant skin lesion should have a maximum 62-day wait time [13]. Surgical wait times were also defined in Australia based on the classification of either malignant or non-malignant, whereby the maximum wait time for each type from the time of consent to surgery is 30 days and 365 days, respectively [14]. In Ontario, wait times for melanoma surgery in Ontario will be instituted in alignment with other Ontario wait time recommendations: 4 weeks from the time of consent to definitive surgery [28]. 

Other jurisdictions have developed quality metrics for skin cancer. In the U.K., the NICE developed and published a set of evidence-based peer-reviewed guidelines and seven quality metrics to standardize the unique aspects of skin cancer care across the NHS [29]. Examples of these quality standards include the following: people with pigmented skin lesions undergoing a specialist assessment have an assessment using dermoscopy and patients with a stage IB–IIC melanoma with a Breslow thickness of more than 1 mm having a discussion about the advantages and disadvantages of a sentinel lymph node biopsy [12]. In Ontario, common quality indicators (length of stay, return to the emergency room, 30- and 90-day mortality) will be reported in 2020 for skin cancer in the Surgical Quality Index Report. Skin-cancer-specific quality indicators will be developed over the next few years with the input of the Skin CAC and OH (CCO)’s Provincial Programs.

## 5. Conclusions

In summary, we have developed unique definitions for complex and non-complex skin cancer and associated clinical services, as well as a skin cancer pathways map. Going forward, our work will include supporting the development of a network of skin cancer biopsy clinics to ensure timely access to diagnosis and treatment, skin-cancer-specific quality indicator development, and the creation of clinical guidelines that are specifically centered around Merkel cell carcinoma and KCs. 

## Figures and Tables

**Table 1:** Ontario Health (Cancer Care Ontario)’s Skin Cancers Advisory Committee definitions.

Complex Skin Cancers
General Factors That Are Applicable to All Types of Skin Cancer
**Patient factors:** Inoperable (patient or tumor factors) Initial assessment for skin cancers associated with genetic mutations (e.g., Gorlin’s syndrome) **Tumor factors:** Node-positive (micro and macro) Locally advanced skin cancers (e.g., large size, involving muscle or bone) Metastatic Subtypes: mucosal melanoma and ocular melanoma Cancers that developed in a scar, burn, or a site that was previously treated In-transit, satellite disease, or recurrent disease **Treatment factors:** Any patient whose treatment requires: Surgical treatment, including lymph node dissection (modified or radical neck dissection, axillary level 1–3 dissection, superficial and deep groin dissection), or resection of metastatic disease A medical oncologist’s opinion A radiation oncologist’s opinion Multidisciplinary care Consideration for clinical trials Mohs micrographic surgery at a Mohs left as per Mohs’s guidelines
**Melanoma** See general factors for stages IIB–IV
**Merkel** All Merkel cell carcinomas
**Squamous Cell Carcinoma** Squamous cell carcinomas that show rapid growth (i.e., within weeks) Histologic features: any of depth > 6 mm, perineural invasion ≥ 0.1 mm, sensory or motor deficits, poorly differentiated, or level IV/V invasion (muscle/bone invasion)
**Basal Cell Carcinoma** Basal cell carcinomas that show rapid growth (i.e., within weeks) Histologic features: perineural invasion, sensory or motor deficits, and level IV/V invasion (muscle/bone invasion)
**Any Other Skin Cancer Histology** Due to their rare occurrence, any skin cancer that is non-melanoma, non-basal cell carcinoma, non-squamous cell carcinoma (i.e., sebaceous gland carcinoma, adnexal tumors, etc.) is considered complex.
**Non-Complex Skin Cancers**
**Melanoma** Stage IA, IB, or IIa cutaneous melanoma
**Merkel** None
**Squamous Cell Carcinoma (SCC)** Any other SCC features not indicated in the complex SCC characteristics
**Basal Cell Carcinoma (BCC)** Any other BCC features not indicated in the complex BCC characteristics

**Table 2:** Clinical services that are required for the management of complex skin cancers.

**Melanoma and** **Merkel cell carcinoma**	**Institution:** Timely diagnosis of suspicious lesions (i.e., does your institution provide on-site biopsies for potential melanomas vs. requiring a diagnosis of melanoma/skin cancer prior to visiting a cancer left/hospital?) Surgical dermatopathology and cytopathology service Molecular testing for melanoma (BRAF/NRAS/ckit) Multi-disciplinary clinics PET, MRI, and CT scans Treatment for in-transit metastases from melanoma other than surgical excision (IL2 or other treatment) Tumor boards (Multidisciplinary Cancer Conference (MCC)) for melanoma and/or skin cancer Clinical trials **Core Services:** Dermatology Plastic surgery Surgical oncology/general surgery Medical oncology Radiation oncology ENT/H&N surgeon Dermatopathology Radiology (including nuclear medicine) Lymphedema care Access/drug facilitator Specialized nursing **Access to:** Palliative care Genetics Vascular or orthopedic surgery (for amputation) Neurosurgery Gyne-oncology Ophthalmology Physicians to treat immunotherapy complications → gastroenterology, ophthalmology, endocrinology, rheumatology, neurology, nephrology, cardiology, and respirology **Allied healthcare providers:** Psychosocial care provider (social worker, psychiatrist, psychologist) Physiotherapist (PT) Occupational therapist (OT) Oncology pharmacist Cosmetic camouflage
**BCC**	**Node-positive and/or metastatic or locally advanced (see above services)** Mohs surgery Capacity for excision with an intraoperative frozen section assessment
**SCC**	**Node positive and/or metastatic or locally advanced (see above services)** Mohs surgery Capacity for excision with an intraoperative frozen section assessment

CT: Computerized Tomography, ENT: Otolaryngology, H&N: Head and Neck, IL2: Interleukin-2, MRI: Magnetic Resonance Imaging, PET: Positron Emission Tomography.

## Data Availability

No new data were created or analyzed in this study. Data sharing is not applicable to this article.

## Abbreviations

BCC	Basal cell carcinoma
CAC	Cancer Advisory Committee
DPM	Disease Management program
KC	Keratinocyte carcinoma
LHIN	Local Health Integration Network
MCC	Multidisciplinary Cancer Conference
MDC	Multidisciplinary Clinic
MeSH	Medical Subject Heading
MMS	Mohs micrographic surgery
NCERG	National Cancer Expert Reference Group
NHS	National Health Service
NICE	National Institute for Health and Care Excellence
OCL	Ontario Cancer Lead
OH (CCO)	Ontario Health (Cancer Care Ontario)
RCC	Regional Cancer Centre
RCP	Regional Cancer Program
RVP	Regional Vice President
SCC	Squamous cell carcinoma
SLNB	Sentinel lymph node biopsy
U.K.	United Kingdom

## Appendix A

**Table A1:** Search strategy for the systematic literature review.

**Date:** 8 November 2018 **Subject:** Complex vs. non-complex melanoma—standard of care/ treatment approach **Date range:** 2015–2018 **Language(s):** English **Database(s):** Medline			
**Search Strategy**			
**Set 1: Complex vs. non-complex skin cancer**			
Exp “melanoma”/ (((complex or malignant) and (cutaneous or skin) and (cancer or neoplasm or tumor or tumour or tumors or tumours or melanoma or “basal cell carcinoma” or BCC or SCC or “Squamous cell carcinoma” or mole or freckle))). ti, ab, kw AND Exp “skin neoplasms”/ (((“non-complex” or “non-malignant”) and (cutaneous or skin) and (cancer or neoplasm or tumor or tumour or tumors or tumours or “non-melanoma” or NMSC or “basal cell carcinoma” or BCC or SCC or “Squamous cell carcinoma” or mole or freckle))). ti, ab, kw AND (definition or define or defining characteristic or clinical characteristic). ti, ab, kw			
**Set 2: Standard of Care**			
Exp “standard of care”/ or ((“standard-of-care” or approach) and (treatment or biopsy or biopsies or diagnosis or prevent)). ti, ab, kw			
**Search History**			
Ovid MEDLINE(R) and Epub Ahead of Print, In-Process, and Other Non-Indexed Citations, Daily and Versions(R) <1946 to 8 November 2018>			
#	**Searches**	**Results**	**Type**
1	Exp “melanoma”/	88,121	Advanced
2	((complex or malignant) and (cutaneous or skin) and (cancer or neoplasm or tumor or tumour or tumors or tumours or melanoma or “basal cell carcinoma” or BCC or SCC or “Squamous cell carcinoma” or mole or freckle)). ti, ab, kw	33,639	Advanced
3	1 or 2	113,386	Advanced
4	Exp “skin neoplasms”/	116,776	Advanced
5	((“non-complex” or “non-malignant”) and (cutaneous or skin) and (cancer or neoplasm or tumor or tumour or tumors or tumours or “non-melanoma” or NMSC or “basal cell carcinoma” or BCC or SCC or “squamous cell carcinoma” or mole or freckle)). ti, ab, kw	207	Advanced
6	4 or 5	116,907	Advanced
7	3 and 6	42,653	Advanced
8	(definition or define or defining characteristic or clinical characteristic). ti, ab, kw	383,531	Advanced
9	7 and 8	852	Advanced
10	Exp “standard of care”/	2865	Advanced
11	((“standard-of-care” or approach) and (treatment or biopsy or biopsies or diagnosis or prevent)). ti, ab, kw	542,482	Advanced
12	10 or 11	544,785	Advanced
13	9 and 12	51	Advanced
14	limit 13 to English language	48	Advanced
15	limit 14 to yr = “2015-Current”	9	Advanced

## Appendix B

**Table A2:** Access to initial diagnosis and diagnosis services for skin cancer care in Ontario by Local Health Integration Network (LHIN).

LHIN	On-Site Biopsies	Molecular Testing			Excision with Frozen Section	SLNB	Complex Lymph Node Dissections				MDC	MCC	Onsite Clinical Trials	Access to Clinical Trials
		BRAF	NRAS	KIT Mutation Analysis			Axillary Level I–III	Superficial Groin	Deep Groin	Neck Dissection				
**1**														
**2**														
**3**														
**4**														
**5**														
**6**														
**7**														
**8**														
**9**														
**10**														
**11**														
**12**														
**13**														
**14**														

Green color: Full access within the LHIN; yellow color: Limited access with the LHIN—some referrals to outside LHIN (blue converted to yellow); grey color: Did not respond; SLNB: sentinel lymph node biopsy, MDC: Multidisciplinary Clinic, MCC: Multidisciplinary Cancer Conference.

**Table A3:** Access to Specialist Service for Skin Cancer Care in Ontario by LHIN.

LHIN	Dermatology	Dermato-Pathology	Plastic Surgery	General Surgery	Surgical Oncology	Medical Oncology	Radiation Oncology	H&N/ENT Surgery	Neuro-Surgery	Nuclear Medicine		Lymph-Edema/ Physio Care	Drug Access Facili-Tation
										Sentinel Node Lymphangiography	PET Scans		
**1**													
**2**													
**3**													
**4**													
**5**													
**6**													
**7**													
**8**													
**9**													
**10**													
**11**													
**12**													
**13**													
**14**													

Green color: Full access within the LHIN; yellow color: Limited access with the LHIN—some referrals to outside LHIN (blue converted to yellow); grey color: Did not respond; ENT: Otolaryngology, H&N: Head and Neck, PET: Positron Emission Tomography.
